# Dynamic Triple-Mode Sorption and Outgassing in Materials

**DOI:** 10.1038/s41598-017-03091-3

**Published:** 2017-06-07

**Authors:** Hom N. Sharma, Stephen J. Harley, Yunwei Sun, Elizabeth A. Glascoe

**Affiliations:** Lawrence Livermore National Laboratory, Livermore, California 94550 United States

## Abstract

Moisture uptake and outgassing can be detrimental to a system by altering the chemical and mechanical properties of materials within the system over time. In this work, we conducted isotherm experiments to investigate dynamic moisture sorption and desorption in markedly different materials, i.e., a polymeric material, Sylgard-184 and a ceramic aluminosilicate material, Zircar RS-1200, at different temperatures (30 °C–70 °C) by varying the water activity (0.0–0.90). Sylgard-184 showed a linear sorption and outgassing behavior with no-hysteresis over the entire temperature and water activity range considered here. Whereas, the sorption and outgassing of Zircar RS-1200 was highly non-linear with significant hysteresis, especially at higher water activities, at all temperatures considered here. The type of hysteresis suggested the presence of mesopores in Zircar RS-1200, whereas the lack of hysteresis in Sylgard-184 indicates that it has a nonporous structure. A diffusion model coupled with a dynamic, triple-mode sorption (Langmuir, Henry, and pooling modes) model employed in this study matched our experimental data very well and provides mechanistic insight into the processes. Our triple-mode sorption model was adaptive enough to (1) model these distinctly different materials and (2) predict sorption and outgassing under conditions that are distinctly different from the parameterization experiments.

## Introduction

Vapor sorption and outgassing phenomena in materials pose a great scientific and technological interest due to their enormous impact on material functionality, compatibility, and stability. Sorption-diffusion processes play a crucial role in many applications such as electronic devices, gas mixture membrane separation, drug delivery, barrier structures for food packaging, and environmental resistance for polymeric composites^1–5^. For example, unintended leaching from or uptake into implantable polymeric medical devices are a serious health concern^6^. Further, materials are sensitive to changes in their environment which is responsible for alteration of mechanical and chemical properties^2, 4, 7^. Such changes are often associated with many undesirable effects such as degradation, chemical incompatibility, and system failure. The synergistic effects of temperature, stress and sorbed solvents often induce material compatibility issues. For long term material storage, moisture uptake/outgassing may be associated with a series of chain reactions which can cause detrimental impact on material integrity. In such scenarios, prior knowledge of the moisture uptake and outgassing properties of materials not only allows for the selection of suitable materials but also the predictions the future performance. Thus, a complete understanding of material specific sorption-diffusion and the ability to predict long term outgassing behavior are crucial.

Historically, sorption-diffusion and outgassing experiments and models in polymeric and non-polymeric materials were limited to a small range of moisture or humidity levels. Specifically, prior modeling efforts have been limited to single relative humidity (RH) step studies; fitting of such data was restricted to a simple diffusion model, such as the the Crank model^8–11^. In contrast, the sorption kinetics understanding and modeling spans a wide variety of equilibrium isotherm models such as Langmuir, Freundlich, Brunauer–Emmett–Teller, Redlich–Peterson, Dubinin–Radushkevich, Temkin, Toth, Koble–Corrigan, Sips, Khan, Hill, Flory–Huggins and Radke–Prausnitz^12^. Many have implemented a ‘dual-mode’ model utilizing Langmuir and Henry’s type sorption modes in non-polymeric and polymeric materials including glassy polymers^1, 3, 13–18^. This classic dual-mode treatment assumes that there are two populations of molecules in the material; i.e., the Henry’s law population, which is dissolved in the dense portion of the polymer; and the Langmuir population, which resides in preexisting excess free volume or ‘holes’^19, 20^. However, such studies and dual-mode treatments were limited to static models and thus lack the real world applicability to dynamic changes in humidity and temperature. The dual-mode model also fails to capture or consider material specific distinct sorption behavior at high relative humidities.

Many materials, especially at high water activity, show a unique sorption behavior, where water molecules start to aggregate leading to an elevated moisture uptake^2, 21, 22^. This clustering or pooling sorption mode is significantly different from Henry’s and Langmuir type sorption and the sorption dynamics cannot be captured by the ‘dual-mode’ sorption model discussed above. Some attempts have been made to utilize Zimm-Lundberg^23, 24^ clustering function to study water clustering mode in materials^22, 25–27^; however, the model is moderately successful at capturing the equilibrium data when clustering occurs, but unable to capture the dynamic data and is not a direct measurement of dynamic water clustering in materials^22^. Although useful, simple diffusion-only models and the Zimm-Lundberg clustering model are not sufficient for the complex system involving multi-component gas/vapor environment along with the multi-material system. Furthermore, prediction of outgassing for multi-material assemblies is even more challenging due to the existence of hysteresis in many materials. Hence, a model sophisticated enough to predict the aging of the assembly, and, specifically, long term compatibility issues of multiple materials in contact with each other and with the surrounding environment is essential.

Within this context, the present contribution explores this multidimensional problem of material-specific sorption-diffusion behavior in a wide range of relative humidities and temperatures and advances our high fidelity model capable of predicting the sorption and outgassing under realistic environmental conditions^4, 21^. Gravimetric type dynamic vapor sorption (DVS) experiments were employed as a convenient way to measure the moisture uptake and outgassing by the samples when exposed to specified water activity levels. The DVS curves were analyzed and modeled to unravel the sorption and desorption mechanisms or modes. The gravimetric curves reveal modes of moisture sorption, coupled with diffusion, which either obeys a Fickian model or deviates resulting in non-Fickian diffusion phenomena. The triple mode sorption-diffusion model^21^ including Henry’s law absorption, Langmuir kinetics adsorption and pooling mode for the clustering along with the Fickian diffusion model was considered to understand the complex material specific sorption-diffusion behavior. Two distinctly different materials were characterized and modeled in this study in order to validate the range of applicability of the model. Sylgard-184 is a commercially available polydimethyl siloxane elastomer and Zircar RS-1200 is fiber-reinforced alumina silica composite. Our analysis shows that for some ‘simple’ materials (e.g., Sylgard-184), a model with a single sorption mode may be enough to capture most of the water activity range; however, such models fail in many other materials (e.g., Zircar RS-1200) and our dynamic triple mode model is needed. The dynamic nature of the diffusion-sorption phenomenon is captured using the transient Langmuir kinetics rather than equilibrium mode. Model parameters are calibrated and optimized using PSUADE code and shuffled complex evolution ﻿(SCE) algorithms.

## Results

### Material specific sorption

**Figure 1:**
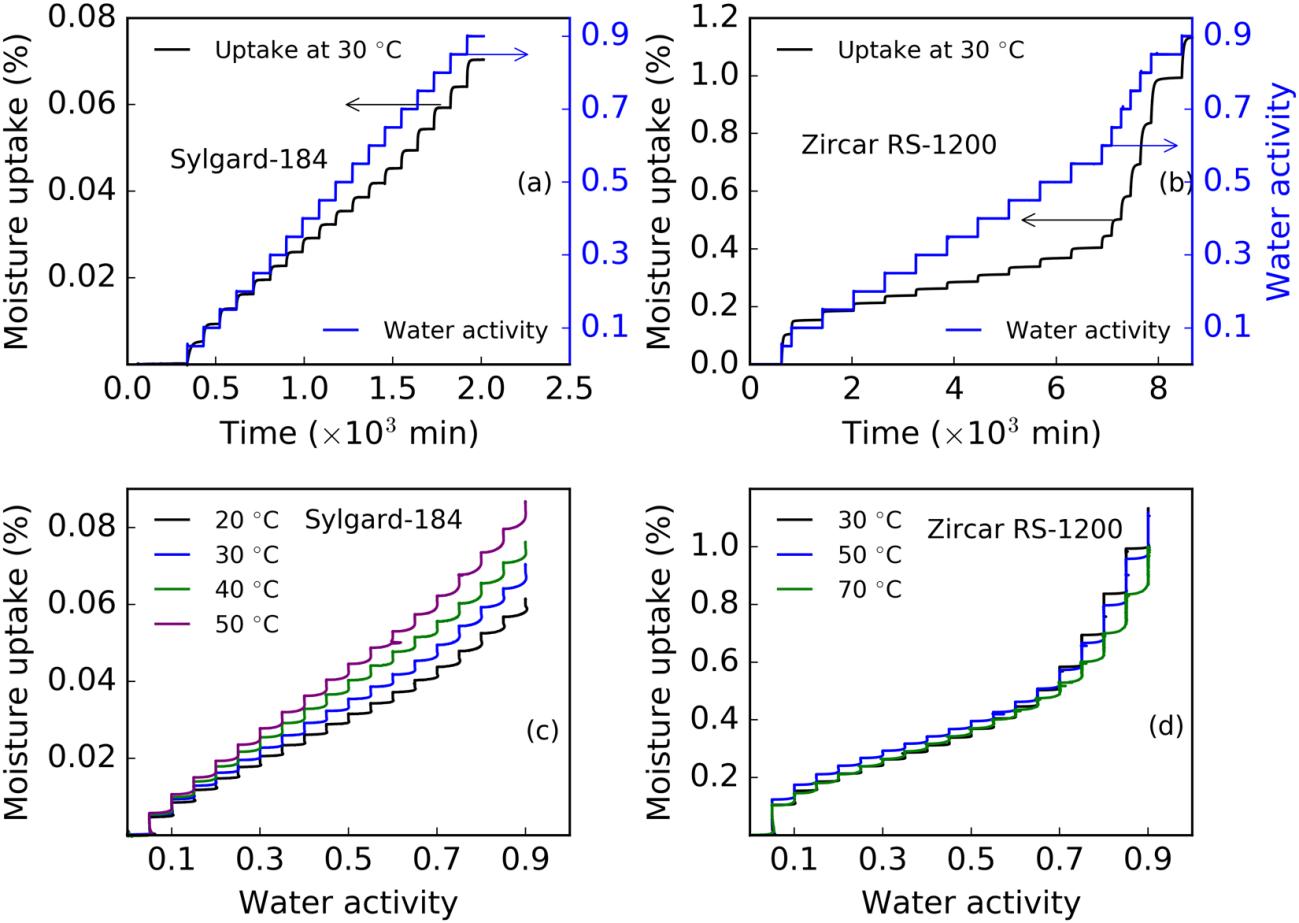
Typical moisture uptake (in %) vs. time (in minutes) or water activity profile for Sylgard-184 and Zircar RS-1200. Panel a: moisture uptake (in %) vs. time for Sylgard-184 at 30 °C, Panel b: moisture uptake (in %) vs. time for Zircar Rs-1200 at 30 °C, Panel c: moisture uptake (in %) vs. water activity for Sylgard-184 at 20, 30, 40, and 50 °C, Panel d: moisture uptake (in %) vs. water activity for zircar RS-1200 at 30, 50, and 70 °C. Water vapor activity from 0 to 0.9 corresponds to the relative humidity range of 0 to 90%.

Figure 1 shows typical experimental moisture uptake profiles obtained from our sorption-diffusion experiments. In the experiment, the water activity was stepped up incrementally from 0 to 0.9 in intervals of 0.05. At each water activity, one can see the dynamic moisture uptake that rises quickly and eventually reaches equilibrium (see panels a and b, Fig. 1). Our investigation showed that the equilibrium moisture sorption behavior can be linear or non-linear and depends on the material. The PDMS material, Sylgard-184, has a linear nature of sorption at all water activities ranging from dry to almost fully saturated moisture conditions (water activity of 0 to 0.9) considered here as shown in Fig. 1a. However, the ceramic type material, Zircar RS-1200, showed three distinct regions (i.e., water activity <0.1 with fast moisture uptake region, water activity <0.65 with linear moisture uptake region, and water activity >0.65 with highly non-linear moisture uptake region) as shown in Fig. 1b over the range of water activity considered. Moisture uptake in the region above the water activity of ~0.65 was significantly different compared to the lower water activity moisture uptake. At the low water activity, the initial uptake was much faster and the rate slowed down until the water activity of ~0.65. We note that moisture uptake by Sylgard-184 was less than one tenth of the uptake by Zircar RS-1200 at the similar conditions. The relatively large uptake by Zircar RS-1200 can be attributed to the presence of an alumina ceramic matrix, which is known to be hydrophilic^7, 28–30^.

The non-linear behavior is distinct in Fig. 1 (panel d) as the moisture uptake percentage is plotted against water activity at multiple temperatures. Each plot has a curved moisture uptake as a function of activity, until it reaches a maximum, which corresponds to the equilibrium moisture uptake at that moisture activity level, then the humidity in the experimental chamber is increased again and the next curved moisture uptake begins. In Sylgard-184, the equilibrium moisture uptake increased linearly with the water activity (see Fig. 1c) and with temperature (from 20 °C to 50 °C), whereas in Zircar RS-1200, the equilibrium moisture uptake was non-linear and showed no significant correlation with temperature (from 30 °C to 70 °C). This suggests that the moisture uptake mechanism in Zircar RS-1200 is controlled by a complex mechanism and multiple sorption-diffusion modes as found in our earlier study of a PDMS material that contained hydrophilic silica filler^21^. We note that the Sylgard-184, commonly selected as moisture diffusion barrier because of its hydrophobic nature^31^, may not be the best for a complete protection as it showed some moisture uptake, albeit low, when exposed to a moist environment.

### Parameters for diffusion and triple-sorption model

**Figure 2:**
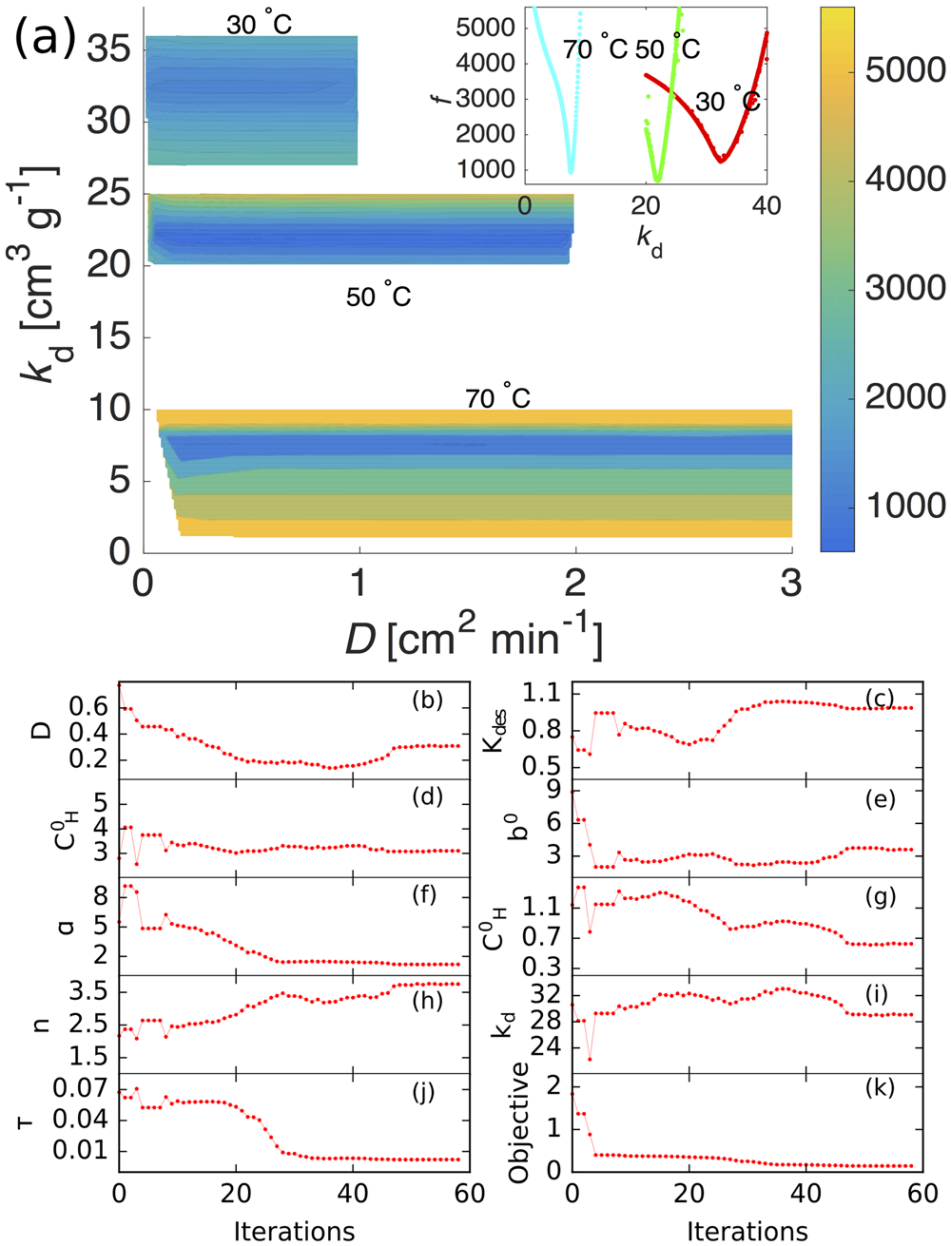
Parameter sensitivity of Henry’s constant (*k*_d_) and diffusivity (*D*), and SCE optimization of parameters for diffusion coupled with triple-mode sorption model. Panel (a) Countour plot showing the sensitivity of Henry’s constant (*k*_d_) and diffusivity (*D*) on the objective function at 30 °C, 50 °C, and 70 °C in Zircar RS-1200. While Henry’s constant is highly sensitive, resulting in a narrow band in blue color in y-axis, diffusivity shows almost no sensitivity at all temperatures considered here. *k*_d_ profiles shown in the inset display a minima for each temperature and illustrate the high sensitivity of the parameter. Panel (b–k): SCE optimization of parameters for diffusion coupled with triple-mode sorption model in Zircar RS-1200 at 50 °C. (**b**): effective diffusivity (*D*, cm^2^ min^−1^), (**c**): desorption rate (k_s_, min^−1^), (**d**): Langmuir capacity (${C}_{{\rm{H}}}^{^{\prime} }$, mg g^−1^), (**e**): Langmuir affinity (*b* ^'^, mg g^−1^), (**f**): pooling factor (*α*), (**g**): pooling threshold (${C}_{{\rm{H}}}^{0}$, mg g^−1^), (**h**): pooling power (*n*), (**i**): Henry’s law constant (*k*_d_, g^−1^ cm^3^), (**j**): reduced tortuosity (*τ*), and (**k**): objective function.

The initial parameter calibration was performed using PSUADE Uncertainty Quantification code^32^. For both materials the calibration was performed using the experimental data at all temperatures considered here. For each parameter set, we generated more than one thousand sample points, which were used to compute and compare the objective function. Our results show that the most sensitive parameter in Zircar RS-1200 is Henry’s law constant (*k*_d_). Figure 2 (panel a) shows the variation in objective function for a range of *k*_d_ and diffusivity (*D*) at three different temperatures in Zircar RS-1200. In the figure, the 70 °C curve has the largest range of diffusivity values and objective functions. The breadth of diffusivity values that result in a local minimum in the objective function indicates that the diffusivity parameter is relatively insensitive. In contrast, the *k*_d_ has a small band of values for each temperature creating a narrow valley region, indicating that a small deviation from the optimal *k*_d_ will result in a significant increase in the value of the objective function.

The calibrated parameters from PSUADE serve as an initial guess for the SCE optimization that was performed to obtain the most precise and accurate model parameters for Sylgard-184 and Zircar RS-1200. The PSUADE and SCE optimization were performed using the only sorption (i.e., uptake only) experimental data at different temperature, the desorption (i.e., outgassing) data was not used in the optimization. Figure 2 (panels, b–k) shows the evolution of parameters during a SCE run for Zircar RS-1200 at 50 °C. Most of the parameters converged after 30–50 iterations as shown in panels b–k, Fig. 2. We note that the computation cost to run these SCE optimizations is very expensive, even for the 1D simulations, and it becomes cost prohibitive for high dimension (i.e., 2D or 3D) problems. Optimized parameters for 50 °C are listed in Table 1. SCE optimized parameters for all other temperatures are provided in Supplementary Information (see Tables S1 and S2).

**Table 1:** SCE optimized parameters for triple-mode sorption in Sylgard-184 and Zircar RS-1200 at 50 °C.

Parameter	Symbol	Calibrated results at 50 °C	
		Sylgard-184	Zircar RS-1200
Effective diffusivity	*D*	1.8221 × 10^−3^	3.0172 × 10^−1^
Desorption rate	*k*_s_	1.0017 × 10^−5^	9.8230 × 10^−1^
Langmuir capacity	${C}_{{\rm{H}}}^{^{\prime} }$	1.1558 × 10^−2^	3.0755
Langmuir affinity	*b*′	9.4430 × 10^−1^	3.7392
Pooling factor	*α*	5.8171 × 10^−1^	1.1869
Pooling threshold	${C}_{{\rm{H}}}^{0}$	6.2288 × 10^−1^	6.2292 × 10^−1^
Pooling power	*n*	1.8398	3.7213
Henry’s law constant	*k*_d_	1.0826 × 10^1^	2.9141 × 10^1^
Reduced tortuosity	*τ*	—	2.0909 × 10^−3^

**Figure 3:**
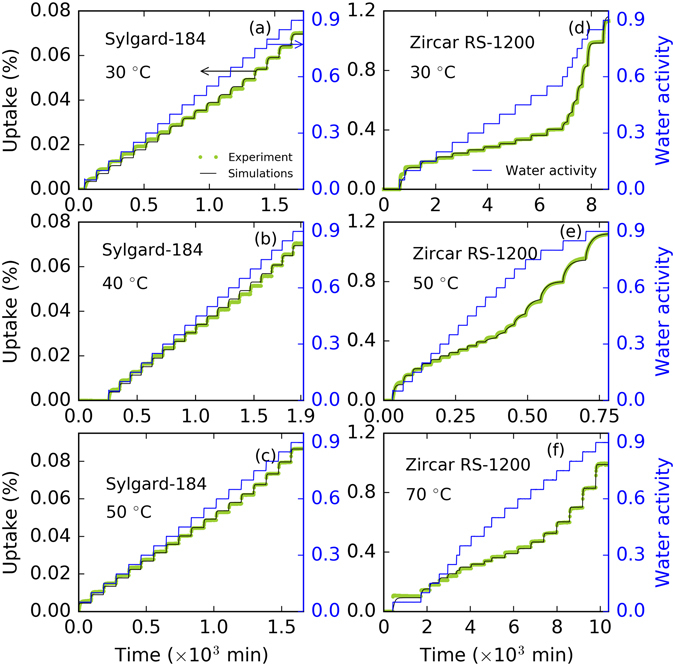
Comparison of the optimized triple-mode sorption-diffusion model against moisture sorption experiments with Sylgard-184 and Zircar RS-1200. Panels (a–c) model performance for Sylgard-184 for uptake (%) vs time (minutes) with water activity in right y-axis at 30 °C, 40 °C, and 50 °C. Panels (d–f) model performance for Zircar RS-1200 for uptake (%) vs time (minutes) with water activity in right y-axis at 30 °C, 50 °C, and 70 °C. Yellow-green symbols, black lines, and blue lines represent the experimental moisture uptake data (on left y-axis), model simulations (on left y-axis), and experimental water activity (on right y-axis), respectively.

With the SCE optimized parameters, we simulated our model using the experimental data obtained at different temperatures. The model simulations for Sylgard-184 and Zircar RS-1200 are shown in Fig. 3. Left panels of Fig. 3(a–c) represent the results for Sylgard-184 and the right panels (d–f) represent the simulation results for Zircar RS-1200. Overall, the model performs well against experimental data over the entire range of water activities considered here. Our statistical analysis of relative error between experimental data and model simulations shows that the probability distribution of error lies between ±3% for Sylgard-184 and ±4% for Zircar RS-1200. Relative error analysis plots are given in the Supplementary Information S1 and S2. The significant difference shown by two materials in the moisture uptake at the same water activity can be related to the parametric space. The most sensitive parameter *k*_d_ is significantly different (see Table 1) in the two materials. At 50 °C, *k*_d_ is ~3 times larger in Zircar RS-1200, which shows how the uptake is influenced by the Henry’s mode. Similarly, the large effective diffusion *D* is associated with the fast moisture diffusion into the material.

**Figure 4:**
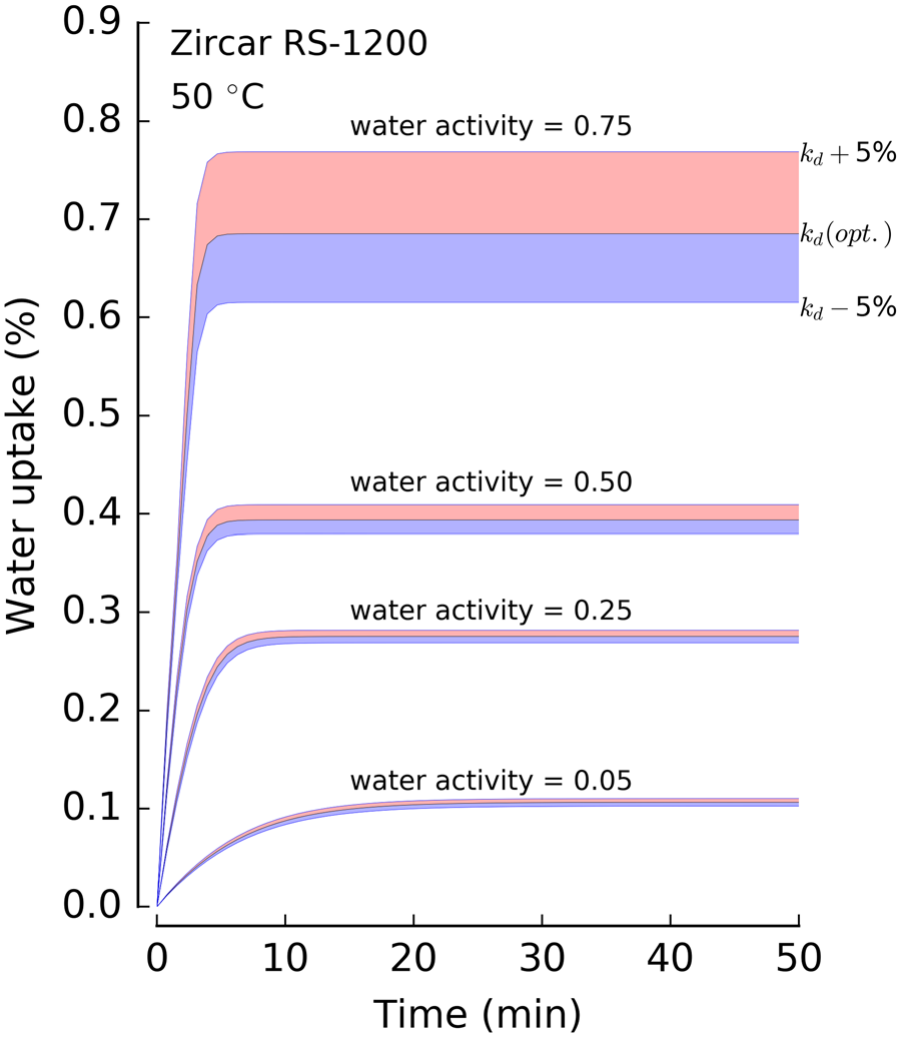
Parameter sensitivity of *k*_d_ at different water activities in Zircar RS-1200 at 50 °C. Water uptake was computed by simulating our model at different water activities (i.e., stepped watter activity from zero level to 0.05, 0.25, 0.50 and 0.75) by perturbing the Henry’s mode constant *k*_d_ by 5% while keeping other parameters constant. The middle line in each water activity region corresponds to the uptake computed with SCE optimized *k*_d_.

Figure 4 displays the parametric sensitivity on water uptake at different water activity regions in Zircar RS-1200 at 50 °C. SCE optimized *k*_d_ was chosen for this analysis. Here we performed a series of simulations in which the sample, which started out at a water activity level of zero, was suddenly exposed to a new water activity level. Optimized *k*_d_ was perturbed by ±5% for each water activity level considered here. Simulation results point out two major phenomena in water sorption by Zircar RS-1200. First, the equilibration time is faster at higher water activity levels. This is due to the increase in water activity gradient between the sample and environment. This further validates the use of our dynamic triple-sorption mode model rather than equilibrium kinetics. Second, the parameter sensitivity is higher at the higher water activity region, which suggests that Pooling mode is important for non-ideal materials and hence validates the need for a model with triple-sorption modes beyond the traditional ‘single’ or ‘dual-mode’ sorption-diffusion models.

### Triple-mode sorption with individual contributions

**Figure 5:**
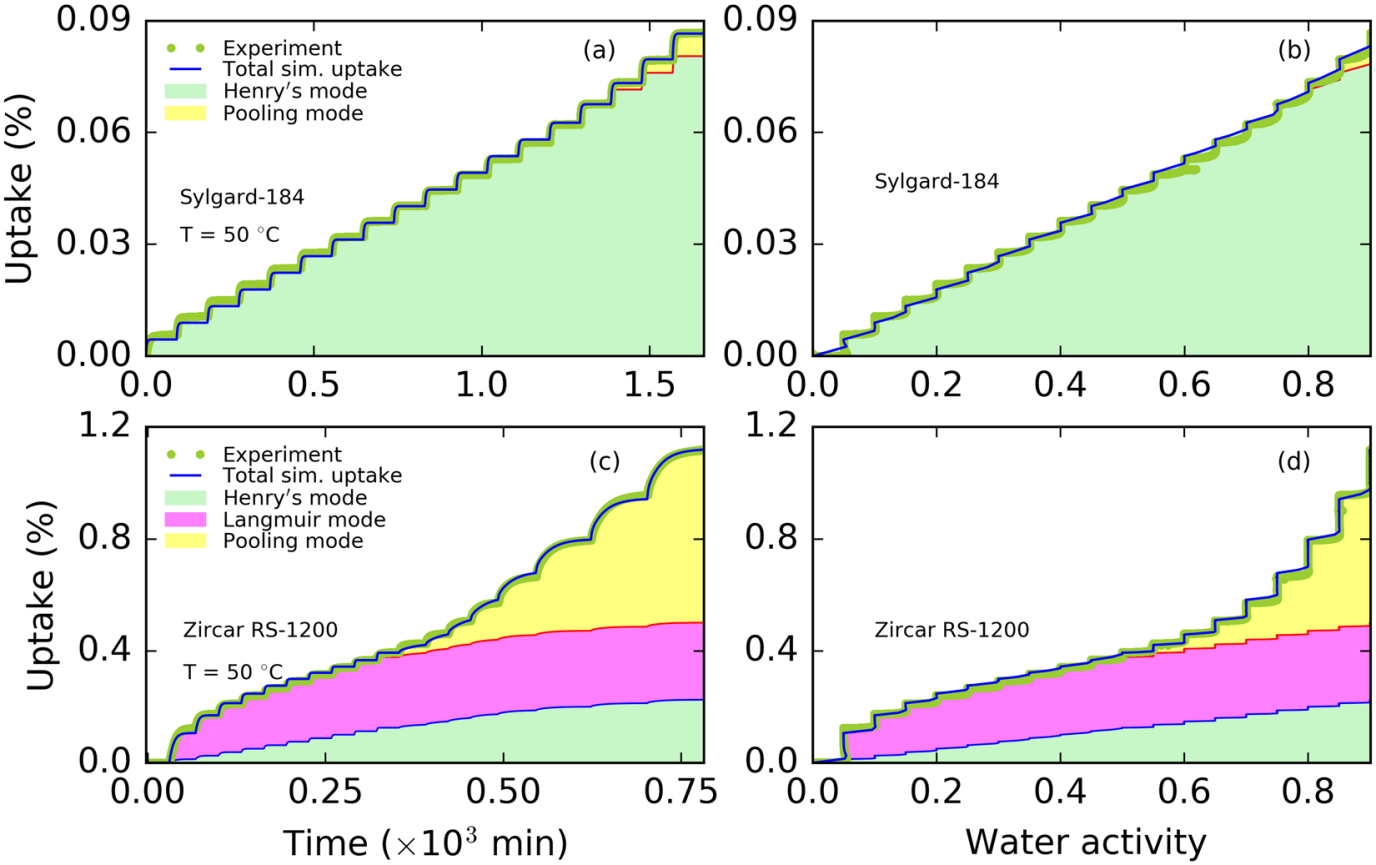
Experimental and simulated mass uptake and individual contributions from triple-mode sorption at 50 °C. Panel a: mass uptake (in %) vs time (in mins × 10^3^) by Sylgard-184, panel b: mass uptake (in %) vs water activity by Sylgard-184, panel c: mass uptake (in %) vs time (in mins) by Zircar RS-1200, and panel d: mass uptake (in %) vs water activity by Zircar RS-1200. Yellow-green symbols represent the experimental data points, blue lines represent the total simulated uptake from triple-mode sorption. Henry’s absorption mode, Langmuir adsorption mode, and pooling sorption mode contributions to the total mass uptake are shown using light green, magenta, and yellow colors, respectively. Sylgard-184 shows almost all uptake due to Henry’s mode of absorption, whereas Zircar RS-1200 shows the contributions from all three modes.

By separating out the individual sorption contributions from the total uptake one can gain insight into the material specific nature of moisture sorption and diffusion. In Fig. 5, we show each contribution to the total uptake, in both materials, at 50 °C. In Sylgard-184 (see Fig. 5, panels a and b), almost all the moisture uptake is due to Henry’s law absorption mode. There is a very small pooling contribution at the highest water activity (~0.9) and no Langmuir adsorption. In contrast, in Zircar RS-1200 all three modes contribute towards the total water uptake creating a distinct pattern over the full range of water activities (see Fig. 5, panels c and d). Notably, at lower water activities, the Langmuir and Henry’s modes dominate sorption. One will notice that Henry’s mode of sorption grows linearly over the entire range whereas the Langmuir mode saturates and reaches an equilibrium above ~0.5 water activity. Pooling sorption mode only becomes active above ~0.5 water activity and increases almost exponentially, which is responsible for the most of the moisture uptake at higher water activities. Moreover, we can clearly identify the sequence of events during the moisture uptake. First, water molecules are adsorbed to the surface sites of the materials due to Langmuir interactions. Then, diffusion and absorption of water into the materials is due to Henry’s mode. Finally, clustering of water inside the material occurs at high water activity giving rise to the pooling effect. We note that the Sylgard-184 did not show Langmuir and Pooling modes of sorption, which is due to lack of hydrophilic filler material (for example, silica filer particles). On the other hand, alumina ceramic matrix and void volume or the porosity plays crucial role in Zircar RS-1200 for the moisture uptake and ultimately to the clustering of water. This type of clustering or self-association of water molecules at higher water activity was also observed in Polylactide materials and filled-PDMS materials^2, 4, 16, 21^.

### Moisture outgassing

**Figure 6:**
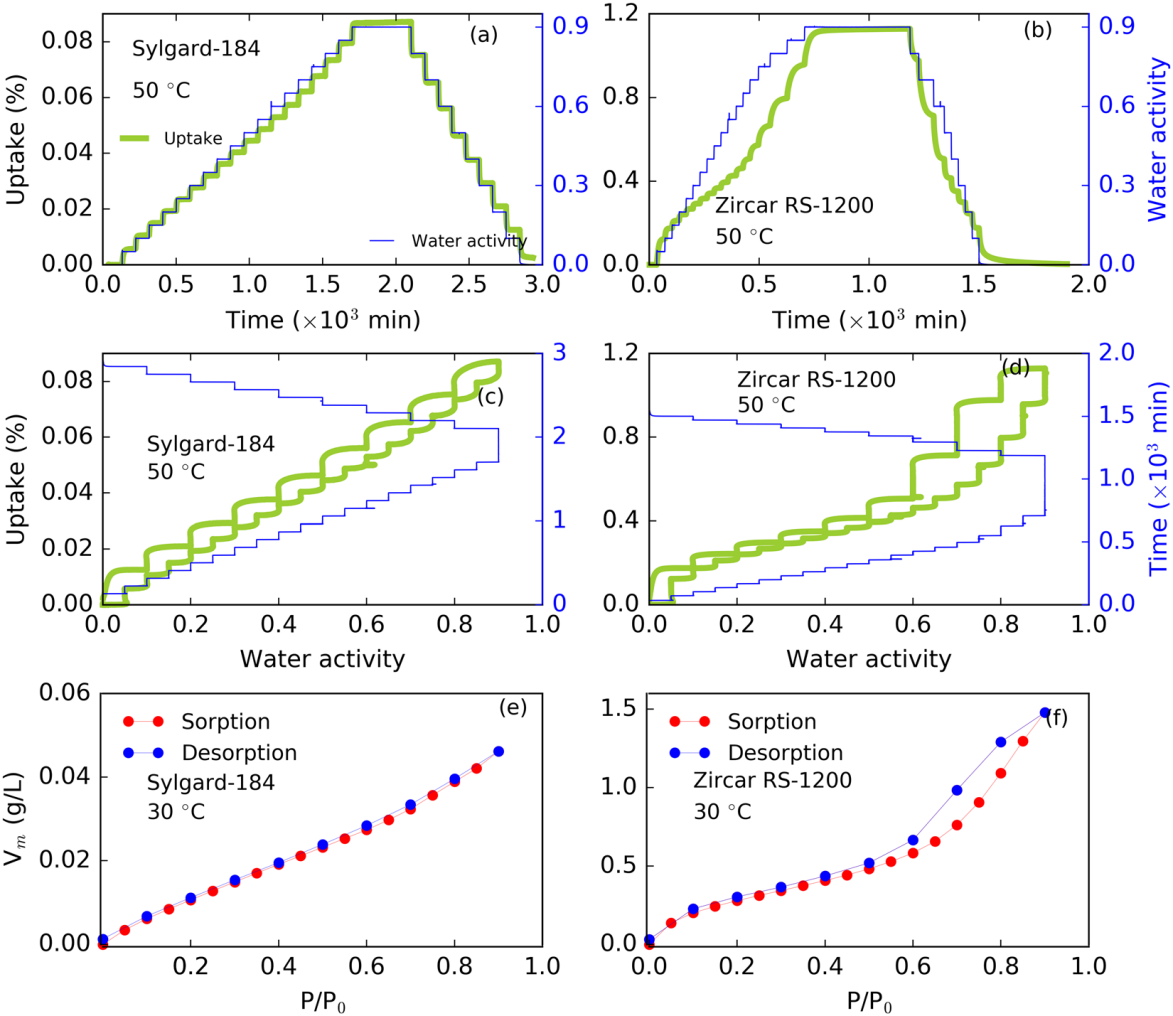
Experimental data from sorption and outgassing at 50 °C, and water vapor sorption and desorption isotherms at 30 °C in Sylgard-184 and Zircar RS-1200. Panel a and b: uptake % as a function of time (in mins) (and water activity in right y-axis) for Sylgard-184 and and Zircar RS-1200. panel c and d: uptake % as a function of water activity (and time in right y-axis) for Sylgard-184 and Zircar RS-1200. Panel e and f: water vapor sorption and desorption isotherms in Sylgard-184 and Zircar RS-1200 at 30 °C. The water vapor activity step is 0.05 in the sorption region, whereas it is 0.1 in the desorption region. Sylgard-184 does not show a hysteresis, whereas Zircar RS-1200 shows a hysteresis between sorption and desorption.

Desorption or outgassing is the opposite of sorption and net outgassing occurs if it is thermodynamically favored. Owing to the interlinked phenomena of moisture sorption and outgassing, understanding of outgassing is equally important in predicting material response in realistic environments. A drop in water vapor activity (i.e., changes in relative humidity), can trigger moisture outgassing if the material is no longer in equilibrium with the surroundings. Here, we studied moisture outgassing from Sylgard-184 and Zircar RS-1200 samples at different water vapor activities. Typically, the samples were taken to the higher water activities (~0.9 or RH = 90%) during sorption steps and allowed to equilibrate, then the water activity was reduced in steps (typically 5–10 RH steps). The complete moisture uptake and outgassing curve for each material at 50 °C is shown in Fig. 6 (Sylgard-184 on left panels and Zircar RS-1200 on right panels). Panel a (in Fig. 6) shows moisture uptake versus time in Sylgard-184. Water vapor activity step changes were 0.05 (RH step 5%) and 0.1 (RH step 10%) during sorption and outgassing, respectively. When uptake and outgassing were plotted together against water activity, the equilibrium uptake amount for each step was in agreement suggesting no prominent hysteresis (see panel c, Fig. 6). With no significant deviation in sorption and outgassing phenomena, one can surmise that Sylgard-184 experiences no change in material structural or morphological properties. In the case of Zircar RS-1200, we can see a significant deviation in uptake versus outgassing, especially at higher water activities, leading to a hysteresis (see Fig. 6, panels b and d). This behavior is indicative of some structural (for example, porosity) change during sorption, which is dominant in the pooling region. In this case, one would not expect Zircar RS-1200’s rigid structure to actually change morphology in these conditions, thus logically the effective porosity must be changing due to clustering of water molecules in the individual pores. This can be further supported by the observations at the lower water vapor activities, where we see the uptake and outgassing curves match. In other words, as the moisture begins to cluster in the pores, the effective porosity in the material changes resulting in slower diffusion rates. This slow diffusion directly affects the rate of outgassing, especially at high water vapor activities.

Figure 6 (panels e and f) shows the sorption and desorption isotherms for Sylgard-184 and Zircar RS-1200 at 30 °C. The molar volume (V_m_) of sorbed water vapor is plotted against relative pressure (P/P_o_). In sylgard-184 (panel e, Fig. 6), the sorption and desorption isotherms are almost identical, suggesting a reversible type (type II) isotherm^33^. In Zircar RS-1200 (panel f, Fig. 6), deviation in isotherm curves can be seen, suggesting an isotherm associated with a hysteresis loop (type IV(a) isotherm^33^). The lack of hysteresis in Sylgard-184 suggests that the material is non-porous. This observation is consistent with the fact that Sylgard-184 does not contain any reinforcing framework in it. Based on IUPAC classification^33, 34^, Zircar RS-1200 shows ‘H2(b)’ type hysteresis, which is due to the complex pore structures. The presence of complex pore structures, often associated with network effect on desorption and various pore blocking mechanism^33^. These phenomena are prevalent in the case where wide pores have access to the external surface only through the narrow necks. Therefore, the wide pores are filled first and remain filled during desorption until the adsorbents from narrow necks get desorbed. This type of hysteresis is often seen in mesocellular silica foams and certain mesoporous silicas after hydrothermal treatment^34, 35^. This is in agreement with our observation of Zircar RS-1200, which contains mesoporous structures with the framework of alumino-silcate matrix. Furthermore, the strong moisture uptake capacity of Zircar RS-1200 is also consistent with our DFT results reported recently, which also suggested a strong surface interaction of moisture and the silica.

### Prediction of moisture outgassing using diffusion triple-sorption model

**Figure 7:**
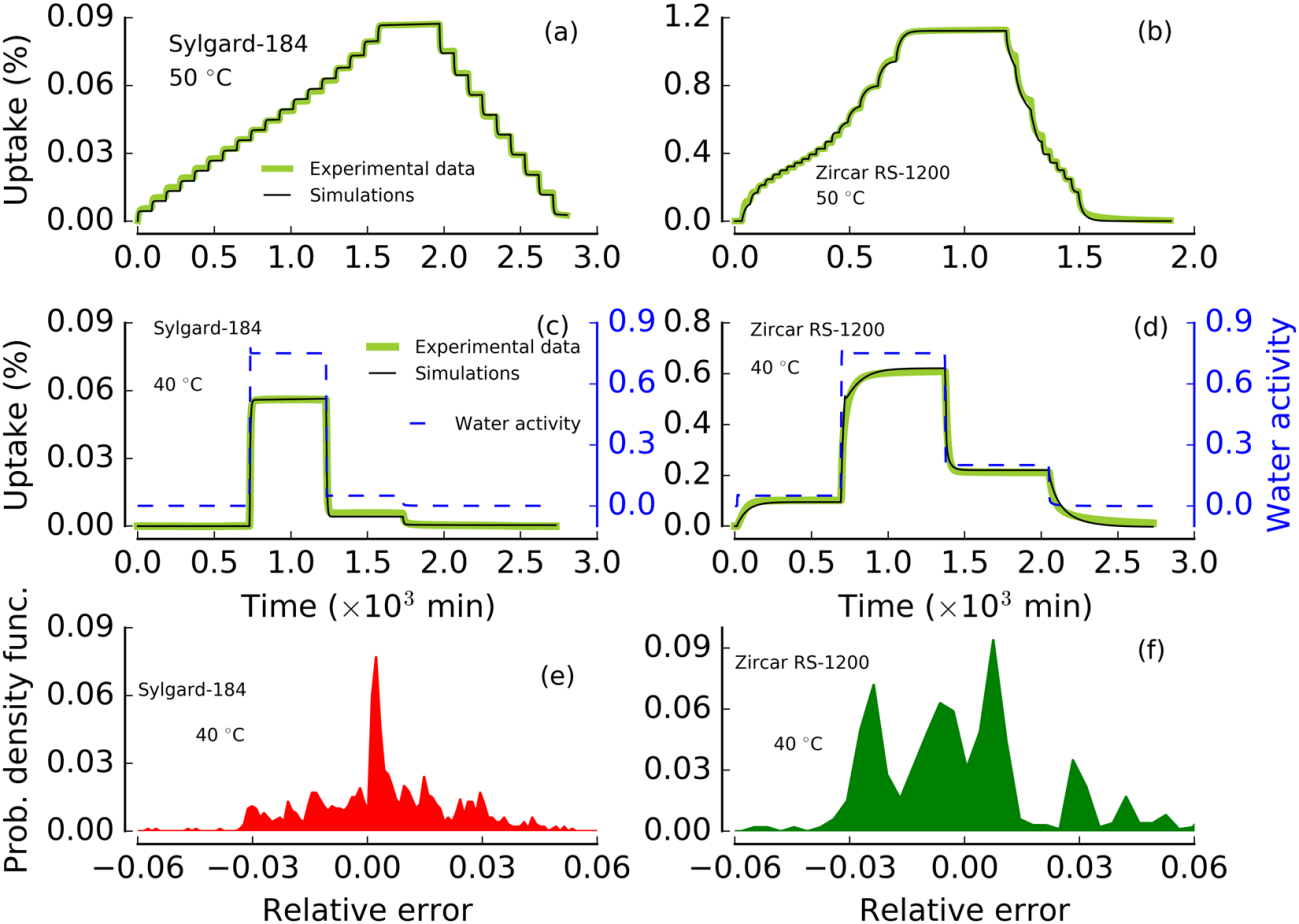
Model performance (for sorption region) and prediction (for outgassing region) in Sygrad-184 (panel a) and Zircar RS-1200 (panel b) at 50 °C. Optimized parameters from sorption region are used to purely predict the outgassing region. Water activity range is 0–0.9 in both experiment with a water activity jump step of 0.05 during sorption and a step of 0.1 during desorption. Similar to sorption, outgassing behavior is distinctly different in Sylgard-184 and Zircar RS-1200 and well captured by our diffusion triple-mode sorption model. Model prediction against step experiments in Sylgard-184 (panel c), and Zircar RS-1200 (panel d) at 40 °C. Water activity is shown on right y-axis in panel c and panel d. Probability density function vs relative error (obtained from relative error between experimental data and model simulations) is shown for Sylgard-184 (in panel e, using data from panel c) and Zircar RS-1200 (in panel f, using data from panel d) at 40 °C.

In this study, we extended our previous triple-sorption model capability to predict the outgassing from the material under realistic conditions. Figure 7 (panel a and b) shows a comparison of experimental data and model simulations and predictions in Sylgard-184 and Zircar RS-1200 at 50 °C. The simulation results for sorption region are same as in Fig. 3, whereas the outgassing/desorption region simulations are pure predictions using SCE optimized parameters. Model predictions for Sylgard-184 (in Fig. 7, panel a) show that moisture outgassing is very similar to the sorption process. This results is in agreement with our observation of ‘no-hysteresis’ in Sylgard-184 (see Fig. 6, panels b and e). Hence, no deviation in parameters is expected. In contrast, Zircar RS-1200 displays a distinct ‘hysteresis’ feature, which shows a slow outgassing at higher water activity region. Our model predictions (see Fig. 7, panel b) are in agreement with the experimental observation within the error margin (≤4%). We can clearly see the smaller pooling effect during outgassing, which could be the main reason for sorption-outgassing hysteresis in Zircar RS-1200. We note that the outgassing prediction in Fig. 7 (panel b) shows minor deviations from the experimental data in the higher water activity outgassing regions. However, the overall fit (pure prediction) is good (see Supplementary Information S4) and our model clearly captures the trend. In general, our diffusion triple-sorption model performs well and predicts the outgassing in distinctly different types of materials.

An ultimate test of the model could be a pure prediction of a whole experimental data with sorption and diffusion process spanning a large step in water activity. To mimic the extreme fluctuations a sample might experience in an application or process (e.g., removal from a dry oven to a humid environment), we conducted an experiment with large jumps in water activity. Figure 7 (panels c and d) shows the experimental results for water uptake vs time (water activity is shown in right y-axis) in Sylgard-184 and Zircar RS-1200. This step-type experiment was conducted by changing water activity from 0 (0% RH) to 0.75 (75% RH) for the sorption region in Sylgard-184, then the water activity was dropped to 0.05 (5% RH) and finally to zero water activity. For Zircar RS-1200, water activity was changed from 0 (0% RH) to 0.05 (5% RH) and to 0.75 (75% RH) for the sorption region, then the water activity was dropped to 0.2 (20% RH) and finally to zero water activity. For Sylgard-184, model parameters from our SCE optimization at 40 °C were used for the prediction, whereas for Zircar RS-1200, average parameter estimate from SCE at 30–70 °C were used. All simulations in panels c and d in Fig. 7 are purely the model predictions without any adjustment in the parameters. Probability density plots for error analysis for Sylgard-184 (using the experimental and modeling data of panel c of Fig. 7) and Zircar RS-1200 (using the experimental and modeling data of panel d of Fig. 7) is shown in panel e and f (Fig. 7), respectively. 95% of the error probability distribution falls within ±4% of error range. Error analysis plots for panels a and b of Fig. 7 are given in Supplementary Information S3 and S4. Our model performs well by predicting the extreme water activity change during sorption as well as outgassing. We note that the model can be equally useful in simulating/predicting sorption and outgassing in other materials.

## Summary and Conclusion

Moisture intrusion rates and capacity in materials, and moisture outgassing from materials are important phenomena that can dictate the lifetime and performance of a material or device. Understanding and quantifying the mechanisms of moisture sorption and diffusion in materials allows for the development of physical-chemical based models to predict a wide range of environments, materials, geometries, and assemblies. Here we have quantified and investigated in two distinctly different materials in order to explore sorption/desorption mechanisms and validate our mechanistic based model. The materials, Sylgard-184 and Zircar RS-1200, were chosen because they are very different from each other, the former is a polydimethyl siloxane elastomer whereas the latter is a highly porous a ceramic aluminosilicate. Experiments were conducted at a range of temperatures (30–70 °C) in isothermal conditions by varying the water activity from 0 to 0.90 (i.e., relative humidities from dry to 90%). Each material had a distinct moisture uptake and outgassing profile with a consistent thermal trend. Whereas Sylgard-184 had a liner uptake and outgassing with no hysteresis, Zircar RS-1200 had a much more complex uptake curve and significant hysteresis in the subsequent outgassing experiment. Total moisture uptake in Zircar RS-1200 was ~10 times larger than the uptake by Sylgard-184 in similar experimental conditions.

The experimental uptake data were used to parameterize a triple-mode sorption and diffusion model. Model simulations match the uptake data at all humidities and temperatures, thus validating and demonstrating the versatility of the model to a wide range of material types. The triple-mode sorption model includes Henry’s absorption, Langmuir adsorption, and pooling mechanisms. In the case of Sylgard-184, modeling efforts demonstrate that a simple Henry’s absorption model is sufficient to accurately and precisely model the moisture uptake between water activities of 0 to 0.85. In contrast, Zircar RS-1200 requires the more sophisticated triple mode sorption model and one can see, via the model simulations, the contributions of each mechanism to the total sorption. The sorption mechanisms are linked to the material physical and chemical properties, specifically the porosity and the hydrophobic or hydrophilic nature of the material.

Outgassing is particularly important to many applications and yet, there is a dearth of literature on predictions of outgassing rates using mechanistically based numerical modeling. Outgassing experiments were performed here in order to fully understand the mechanisms of sorption and to validate our model. As mentioned earlier, each material had a different outgassing profile where hysteresis was observed in some and not others. The main reason for hysteresis is the kinetics of desorption and the rate of diffusion, which are dictated by the chemical and physical nature of the material. Outgassing simulations were executed using the model that was parametrized from uptake data. The model predicted the outgassing for both materials remarkably well. Finally, a third type of experiment type was performed in which the sample was exposed to a few extreme steps in water activity in order to test the model against a more realistic scenario. In this simulation, the model parameters were untouched, thus the simulation is a pure-prediction. The excellent match of simulation to experiment demonstrates the robustness and versatility of the model.

This high fidelity diffusion triple-sorption model can provide insight in to material specific sorption properties and can be applicable to any other materials. We are expanding our modeling capability to perform multi-dimensional simulations and validating the model against 3D samples in order to use the model for more realistic scenarios. These multidimensional simulations and experiments will be presented in a subsequent publication.

## Methods

### Experimental details

All the experiments reported here are conducted using the IGAsorp^36^ instrument, designed by Hiden Isochema, which is equipped with a high resolution micro-balance. The instrument has relative humidity regulation accuracy of 0.02%, weight resolution of 0.05 *μ*g, and temperature regulation accuracy of 0.01 °C. Details on the equipment and experimental setup can be found in prior publications^2, 4^. Two different types of materials, i.e., Sylgard-184 (typical sample dimensions: length = 3.115 cm, width = 1.59 cm, height = 0.119 cm, and density = 0.965 g cm^−3^) and Zircar RS-1200 (typical sample dimensions: length = 2.65 cm, width = 1.074 cm, height = 0.327 cm, and density = 1.986 g cm^−3^), were used for this comparative study. Sample dimensions were measured using high precision calipers from Swiss Precision Instruments, Inc.^37^. Sample density was computed utilizing the sample weight and measured sample volume. Sylgard-184, a polydimethylsiloxane (PDMS) elastomer, samples were prepared in our lab using the technique described elsewhere^2^. Fully cured samples were used for the experiments conducted here. Prior to each experiment in the IGAsorp, each sample was preconditioned with dry nitrogen stream of 250 ml min^−1^ for several days until a stable mass was observed, which allowed us to obtain a true dry state sample and served as a baseline for the sample mass change. Zircar RS-1200, a high strength composite which is a combination of strong reinforcing fibers tightly bound in an alumina ceramic matrix (typically ~82% Al_2_O_3_, ~12% SiO_2_, and ~6% other metal oxides) and designed for use as thermo-mechanical and electrical insulation, samples were obtained from ZIRCAR Refractory Composites, Inc., and machined to the appropriate sample dimensions without the use of water or solvents. Similar preconditioning was performed on the Zircar samples prior to the experiment. The water activity range of 0–0.9 (i.e., Relative humidity range of 0–90%) and temperature range of 30–70 °C were considered in this study. For a typical isotherm sorption/desorption study, the water activity was scanned with 0.05–0.1 step corresponding to 5–10% RH. A few experiments with larger water activity steps (0.2–0.7) were carried out to see the effect of a sudden change of moisture environment on samples. The moisture uptake (in %) is defined as,

1 $$u=\frac{m-{m}_{0}}{{m}_{0}}\times 100$$

where *m*_0_, *m*, and *u* are the initial dry mass, instantaneous moist mass, and the percentage mass uptake, respectively.

### Diffusion-sorption model

The mass balance equation with diffusion, kinetic Langmuir adsorption, Henry’s absorption, and pooling sorption in a material can be written as^21^:

2 $$\frac{\partial C}{\partial t}=\frac{\partial ({C}_{{\rm{H}}}+{C}_{{\rm{L}}}+{C}_{{\rm{P}}})}{\partial t}=\nabla \cdot (D\nabla C)-\frac{d{C}_{{\rm{H}}}}{dt}-\frac{d{C}_{{\rm{P}}}}{dt}-{k}_{{\rm{a}}}SC+{k}_{{\rm{s}}}({C}_{{\rm{H}}}^{^{\prime} }-S)$$

where *C* [mg g^−1^] is the mobile gas concentration in terms of sample bulk mass, *C*_H_ [mg g^−1^] is the mass concentration of the absorbed (i.e., Henry’s mode) gas component per unit of sample bulk mass, *C*_L_ [mg g^−1^] and *C*_P_ [mg g^−1^] are the concentrations in Langmuir and pooling (clustering) modes, respectively, *D* [cm^2^ min^−1^] is the effective diffusion coefficient, and *t* [min] is the time. The last two terms of (2) describe the kinetics of reversible Langmuir adsorption. *k*_a_ [min^−1^ mg^−1^ g] and *k*_s_ [min^−1^] are absorption and desorption rates, *S* [mg g^−1^] is the concentration of empty Langmuir sites, and ${C}_{{\rm{H}}}^{^{\prime} }$ [mg g^−1^] is the Langmuir capacity constant. The Henry’s mode concentration, *C*_H_, is treated as a mobile species due to its linear dependence on gas phase (mobile) concentration.

3 $${C}_{{\rm{H}}}={k}_{{\rm{d}}}\,c={k}_{{\rm{d}}}{\rho }_{{\rm{b}}}C,$$

in which *k*_d_ ∝ *ϕ*/*ρ*_b_ [cm^3^ g^−1^] is Henry’s law constant, *ϕ* is the porosity, *ρ*_b_ [g cm^−3^] is the bulk density, *c* [mg cm^−3^] is the gas-phase concentration of vapor. With the treatment of Henry’s mode as a mobile species, Eq. (2) can be expressed in terms of *C*_H_

4 $$\frac{1}{{k}_{{\rm{d}}}{\rho }_{{\rm{b}}}}\frac{\partial {C}_{{\rm{H}}}}{\partial t}=\nabla \cdot (D\frac{1}{{k}_{{\rm{d}}}{\rho }_{{\rm{b}}}}\nabla {C}_{{\rm{H}}})-\frac{d{C}_{{\rm{H}}}}{dt}-\frac{d{C}_{{\rm{P}}}}{dt}-\frac{{k}_{{\rm{a}}}}{{k}_{{\rm{d}}}{\rho }_{{\rm{b}}}}S{C}_{{\rm{H}}}+{k}_{{\rm{s}}}({C}_{{\rm{H}}}^{^{\prime} }-S\mathrm{)}.$$

When *k*_d_ *ρ*_b_ ≫ 1, Eq. 4 can be written as

5 $$\frac{\partial {C}_{{\rm{H}}}}{\partial t}=\nabla \cdot (De\nabla {C}_{{\rm{H}}})-\frac{d{C}_{{\rm{P}}}}{dt}-{k}_{{\rm{a}}}^{^{\prime} }SC+{k}_{{\rm{s}}}({C}_{{\rm{H}}}^{^{\prime} }-S)$$

where *D*_e_ = *D*/*k*_d_ *ρ*_b_ and $${k}_{{\rm{a}}}^{\text{'}}$$ = *k*_a_/*k*_d_ *ρ*_b_.

In the Langmuir sorption mode, the Langmuir affinity constant b′ (in mg^−1^ g) can be defined as,

6 $$b^{\prime} =\frac{{k}_{{\rm{a}}}}{{k}_{{\rm{s}}}}.$$

The equilibrium pooling concentration, *C*_P_ (in mg g^−1^), is a nonlinear function of Henry’s mode local concentration and is defined as:

7 $${C}_{{\rm{P}}}=\frac{{K}_{{\rm{C}}}^{^{\prime} }{({k}_{{\rm{d}}}{C}_{{\rm{H}}})}^{n}}{n}=\alpha {C}_{{\rm{H}}}^{n}$$

where ${K}_{{\rm{C}}}^{^{\prime} }$ is the equilibrium constant for a clustering reaction, *k*_d_ is the Henry’s law constant, *α* is the lumped pooling coefficient, and *n* represents the number of molecules in each pool and is treated as a fitting parameter at continuum scale. The lumped pooling coefficient *α* is defined as,

8 $$\alpha =\frac{{K}_{{\rm{C}}}^{\text{'}}{k}_{{\rm{d}}}^{n}}{n}$$

The pooling concentration is further expressed as:

9 $${C}_{{\rm{P}}}=\alpha  {\mathcal H} ({C}_{{\rm{H}}},{C}_{{\rm{H}}}^{o}){({C}_{{\rm{H}}}-{C}_{{\rm{H}}}^{o})}^{n}$$

where ${C}_{{\rm{H}}}^{o}$ (in mg g^−1^) is the threshold value of Henry’s concentration, at which the Pooling mode starts. ${\mathcal H}$ is the heaviside step function, which is expressed as^21^:

10 $$ {\mathcal H} =\{\begin{array}{cc}\mathrm{1,} & {C}_{{\rm{H}}} > {C}_{{\rm{H}}}^{o}\\ \mathrm{0,} & {\rm{otherwise}}\end{array}$$

To account for a decreased effective diffusion once Pooling begins, a reduced tortuosity^21^ parameter *τ* was introduced at the point at which pooling started; below this point the tortuosity was 1.0. The effective diffusivity can be calculated as,

11 $$D={D}_{o}\tau $$

where *D* is effective diffusion coefficient, *D*_o_ is molecular-weight-dependent diffusivity, and *τ* is medium-specific tortuosity, which is a measure of the connectivity of pores and defined as the chord-arc ratio (ratio of the straight distance to the integrated length of the tortuous pathway).

### Parameters estimation and optimization

Model parameters are estimated using the uncertainty quantification code PSUADE^32, 38^ and calibrated using the shuffled complex evolution (SCE) method^39^. First, a sampling based non-intrusive Latin Hypercube (LH) sampling method^40^ is used to generate large number of sample points; sufficiently large to represent the parametric space. Each ‘sample point’ consists of a vector of all parameters (i.e., *D*, *k*_s_, ${C}_{{\rm{H}}}^{^{\prime} }$, *b*′, *α*, *n*, and *k*_d_) in our model. To be consistent with equilibrium Langmuir formulation, we use the Langmuir affinity constant *b*′ = *k*_a_/*k*_s_ [mg^−1^ g] instead of *k*_a_ in our parameter calibration. Then, each sample point is used to parametrize the model and the corresponding objective function is computed. Sample points resulting the smallest minimization function are chosen to be the candidates for the parameter optimization using the SCE^39^ method implemented in MATLAB^41^ with lower and upper bounds of all parameters defined. The objective function used for model calibration is:

12 $$f={\int }_{t}|m(t)-\hat{m}(t)|dt$$

in which *m* and $\hat{m}$ are the experimental and simulated mass uptake, respectively. The model simulated mass uptake is calculated as:

13 $$\hat{m}={\rho }_{b}{A}_{0}{\int }_{0}^{L}({C}_{{\rm{H}}}+{C}_{{\rm{L}}}+{C}_{{\rm{P}}})dx,$$

where *ρ*_b_ and *A*_0_ are the bulk sample density and sample area, respectively. SCE in PSUADE is used to find the best fit of model to experimental data with a set of best fit parameters. Our SCE optimization convergence criterion was set to 1 × 10^−5^ for the relative change in the objective function. Once the convergence criterion has been met, the optimization is set to complete and the final parameters are obtained. Our model parameters are set to be accurate within the error margin of 0.01%.

## Electronic supplementary material

Supplementary Information
