# Supplementary material for: Dynamic Triple-Mode Sorption and Outgassing in Materials
Source: Sci Rep. 2017 Jun 7;7:2942. doi: 10.1038/s41598-017-03091-3 (PMC5462788; doi:10.1038/s41598-017-03091-3)

Dynamic Triple-Mode Sorption and Outgassing in Materials

Hom N. Sharma, Stephen J. Harley, Yunwei Sun, and Elizabeth A. Glascoe*

Lawrence Livermore National Laboratory

7000 East Ave, Livermore, California 94550, United States

E-mail: glascoe2@llnl.gov

Table S1: SCE optimized parameters for Sylgard-184 at 30, 40, and 50 °C.

**Sn**	**Parameter**	**Sylgard-184**		
		**30** °C	**40** °C	**50** °C
1	Effective diffusivity (D)	$1.202{\times}10^{-3}$	$1.791{\times}10^{-3}$	$1.822{\times}10^{-3}$
2	Desorption rate (k_s)	$1.092{\times}10^{-4}$	$8.599{\times}10^{-4}$	$1.001{\times}10^{-5}$
3	Langmuir capacity (C'_H)	$1.012{\times}10^{-3}$	$5.539{\times}10^{-2}$	$1.155{\times}10^{-2}$
4	Langmuir affinity (b')	$9.502{\times}10^{-1}$	$1.018{\times}10^{-1}$	$9.443{\times}10^{-1}$
5	Pooling factor (α)	$4.831{\times}10^{-1}$	$4.77{\times}10^{-1}$	$5.817{\times}10^{-1}$
6	Pooling threshold (C_H^o)	$4.851{\times}10^{-1}$	$5.793{\times}10^{-1}$	$6.228{\times}10^{-1}$
7	Pooling power (n)	1.804	2.159	1.839
8	Henry's law constant (k_d)	$2.344{\times}10^{1}$	$1.484{\times}10^{1}$	$1.082{\times}10^{1}$

Table S2: SCE optimized parameters for Zircar RS-1200 at 30, 50, and 70 °C.

Sn	Parameter	Zircar RS-1200		
		30 °C	50 °C	70 °C
1	Effective diffusivity (D)	$2.0142{\times}10^{-2}$	$3.017{\times}10^{-1}$	$2.08{\times}10^{-2}$
2	Desorption rate (k_s)	$6.27184{\times}10^{-1}$	$9.823{\times}10^{-1}$	$8.655{\times}10^{-1}$
3	Langmuir capacity (C'_H)	3.13637	3.075	3.211
4	Langmuir affinity (b')	4.55268	3.739	3.385
5	Pooling factor (α)	5.33525	1.186	1.575
6	Pooling threshold (C_H^o)	$4.02646{\times}10^{-1}$	$6.229{\times}10^{-1}$	$5.487{\times}10^{-1}$
7	Pooling power (n)	3.2268	3.721	4.028
8	Henry's law constant (k_d)	$5.5268{\times}10^{1}$	$2.914{\times}10^{1}$	$1.69{\times}10^{1}$
9	Reduced tortuosity (τ)	$1.23{\times}10^{-2}$	$2.090{\times}10^{-3}$	$5.627{\times}10^{-2}$

Figure S1: probability density plot for error analysis in Sylgard-184. Relative error (computed using $relative\ error = \frac{model\ result - \text{experimental data}}{experimental\ data}$) is relatively small and centered between $\pm 3\%$ indicative of an excellent match between experiment and simulations as shown in figure below. Analysis corresponds to Fig. 3 in the main text.

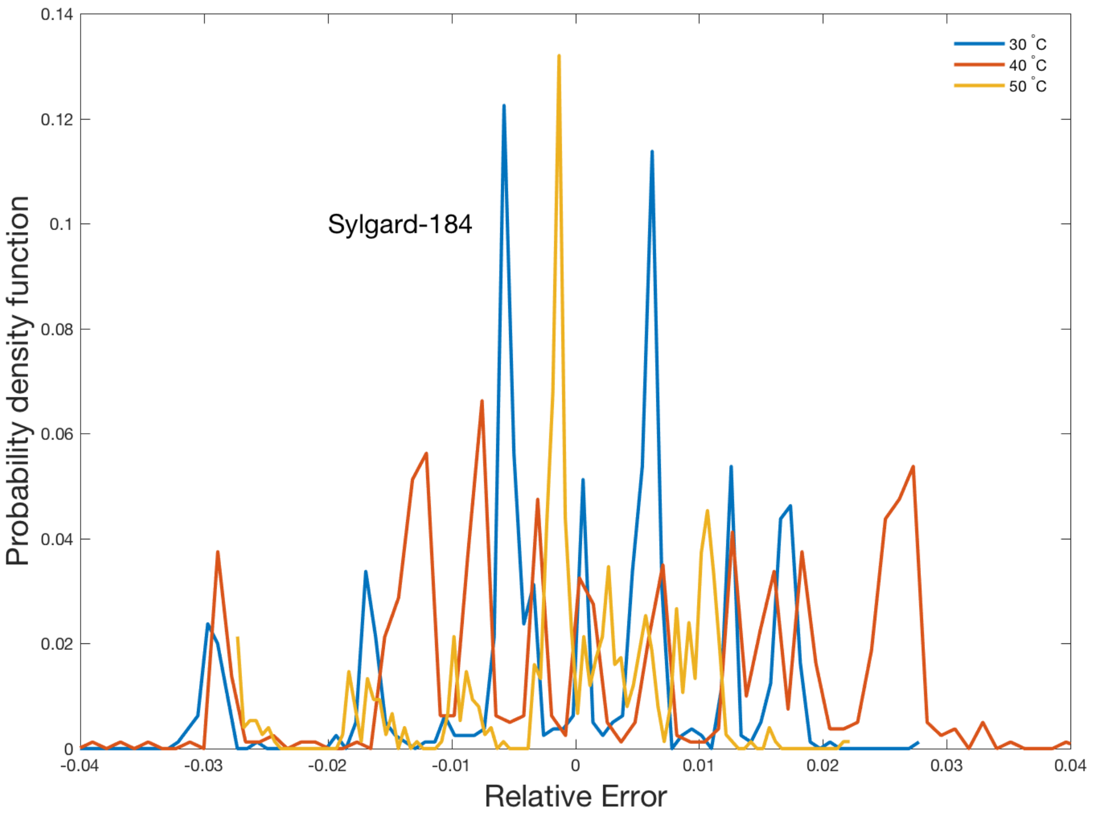

Figure S2: probability density plot for error analysis in Zircar RS-1200 corresponding to Fig. 3 in the main text.

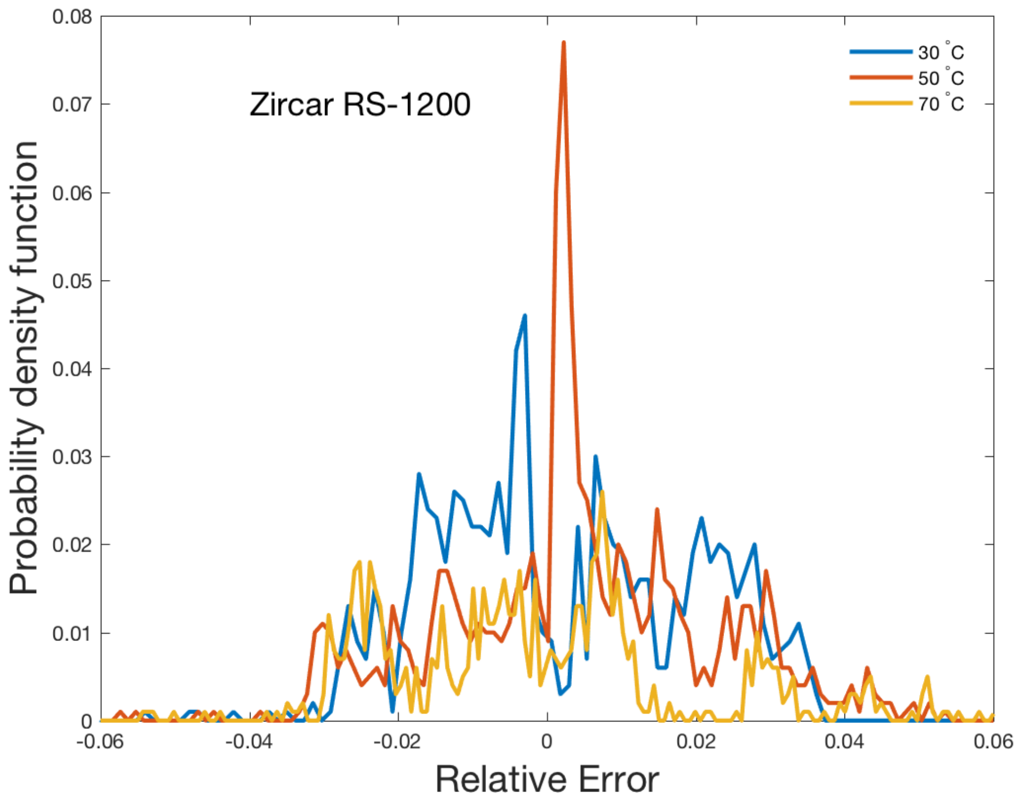

S3: Probability density plot for error analysis in Sylgard-184 at 50 °C corresponding to the experimental data and simulations from Fig 7a in the main text.

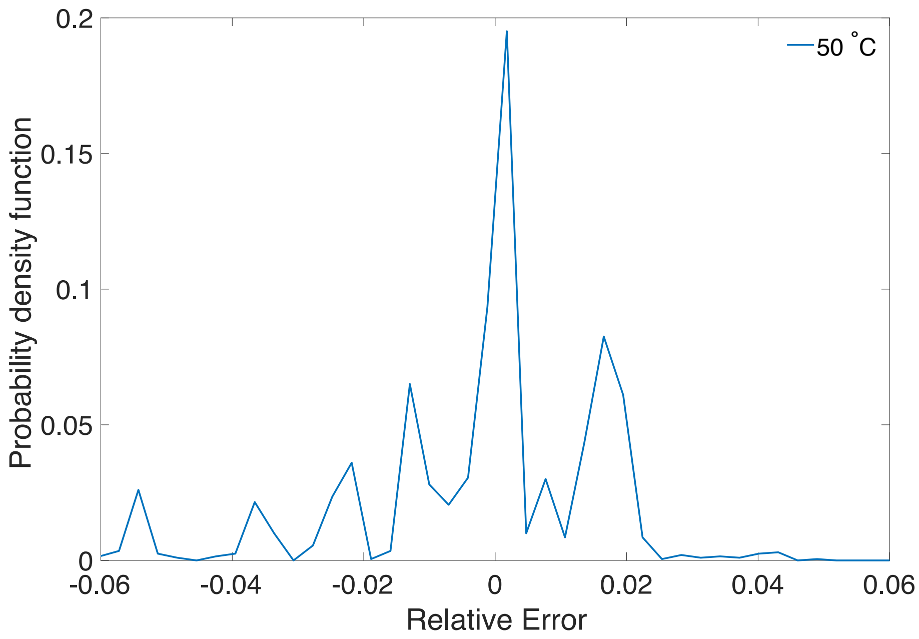

S4: Probability density plot for error analysis in Zircar RS-1200 at 50 °C corresponding to the experimental data and simulations from Fig 7b in the main text.

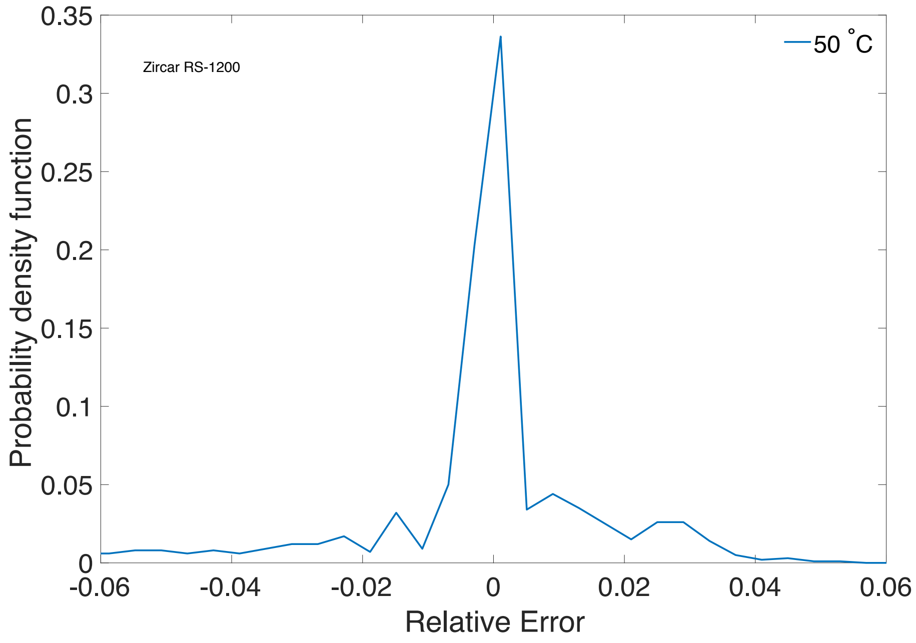

Supplement: Supplementary file 1 — Supplementary Information [file 41598_2017_3091_MOESM1_ESM.pdf]
